# Machine Learning-Based Prediction of Feed Conversion Ratio: A Feasibility Study of Using Short-Term FCR Data for Long-Term Feed Conversion Ratio (FCR) Prediction

**DOI:** 10.3390/ani15121773

**Published:** 2025-06-16

**Authors:** Xidi Yang, Liangyu Zhu, Wenyu Jiang, Yiting Yang, Mailin Gan, Linyuan Shen, Li Zhu

**Affiliations:** 1 State Key Laboratory of Swine and Poultry Breeding Industry, College of Animal Science and Technology, Sichuan Agricultural University, Chengdu 611130, China; yangxidi@stu.sicau.edu.cn (X.Y.); zhuliangyu@stu.sicau.edu.cn (L.Z.); 2022302092@stu.sicau.edu.cn (W.J.); yangyiting0914@stu.sicau.edu.cn (Y.Y.); ganmailin@sicau.edu.cn (M.G.); 2 Key Laboratory of Livestock and Poultry Multi-Omics, Ministry of Agriculture and Rural Affairs, College of Animal Science and Technology, Sichuan Agricultural University, Chengdu 611130, China; 3 Farm Animal Genetic Resources Exploration and Innovation Key Laboratory of Sichuan Province, Sichuan Agricultural University, Chengdu 611130, China

**Keywords:** feed conversion ratio (FCR), machine learning, data prediction, deep learning, precision livestock farming

## Abstract

Feed conversion ratio (FCR) is a critical indicator of production efficiency in livestock husbandry. Improving FCR is essential for optimizing resource utilization and enhancing productivity. Traditional methods for FCR optimization rely on experience and long-term data collection, which are time-consuming and inefficient. This study explores the feasibility of predicting long-term FCR using short-term FCR data based on machine learning techniques. We employed nineteen machine learning algorithms, including Linear Regression, support vector machines (SVMs), and Gradient Boosting, using historical datasets to train and validate the models. The results show that the Gradient Boosting model demonstrated superior performance, achieving a coefficient of determination (R^2^) of 0.72 and a correlation of 0.85 between predicted and actual values when the testing interval exceeded 40 kg. Therefore, we recommend a minimum feeding measurement interval of 40 kg. Furthermore, when the testing interval was set to 40 kg and further refined to the range of 50–90 kg, the model achieved an R^2^ of 0.81 and a correlation of 0.90 for FCR prediction in the 30–105 kg range. Among the 19 machine learning algorithms tested, Gradient Boosting, LightGBM, and CatBoost showed superior performance, with Gradient Boosting achieving the best results. Considering practical production requirements, the 50–90 kg feeding stage proved to be the most ideal for FCR testing. This study provides a more effective method for predicting feed efficiency and offers robust data support for precision livestock farming.

## Simple Summary

In livestock farming, accurately predicting the feed conversion ratio (FCR) is crucial for improving production efficiency and reducing costs. This study aimed to explore whether machine learning could use short-term FCR data to predict long-term FCR. Using 438,552 feed samples from pigs in Sichuan, the researchers tested 19 machine learning algorithms. The results showed that the Gradient Boosting model performed best. When the testing interval exceeded 40 kg, this model achieved high accuracy, with a coefficient of determination of 0.72 and a correlation of 0.85 between predicted and actual values. The most ideal feeding stage for FCR testing was found to be 50–90 kg. These findings provide an effective way to predict feed efficiency, helping farmers optimize feeding strategies, allocate resources better, and manage livestock more proactively, thus contributing to more sustainable and efficient animal husbandry.

## 1. Introduction

With the continuous growth of the global population and increasing resource scarcity, precision agriculture technology has become a key tool for improving agricultural production efficiency [1]. Yamsani et al. [2] developed a systematic framework for using historical farm data to predict future productivity patterns, which integrates IoT sensor networks and machine learning algorithms to monitor livestock behaviors, analyze real-time physiological data, and establish predictive models for optimizing resource allocation and breeding strategies in smart agriculture.

In animal husbandry, the feed conversion ratio (FCR) is a key indicator of production efficiency. Prakash et al. [3] performed an extensive genetic analysis of FCR and related growth parameters in broiler chickens, establishing the hereditary basis for feed efficiency. Davison et al. [4] built upon this by developing machine learning models that predict FCR using feeding behavior data, while France et al. [5] established advanced methods for evaluating animal feed quality and its impact on FCR.

The complexity of FCR optimization has been well documented. Morota et al. [6] created a comprehensive framework for collecting and analyzing feed efficiency data, while Sharma et al. [7] conducted comparative analyses of various machine learning models for predicting livestock productivity. Mahmud [8] explored the challenges and opportunities in applying machine learning to livestock farming, particularly focusing on data quality and model selection issues. Zhang fan et al. [9] demonstrated practical applications by successfully implementing neural networks for FCR prediction in dairy cows.

Recent technological advances have enabled more sophisticated approaches to FCR prediction. Gaillard et al. [10] summarized the techniques and models of precision sow feeding. Zhou and Hooker [11] developed innovative decision tree models with varying coefficients, significantly improving prediction accuracy. Li and Ma [12] showcased the practical implementation of AI techniques in poultry farming, achieving notable improvements in FCR optimization.

The theoretical foundation for these advances was strengthened by Montgomery et al. [13], who provided the statistical framework for regression analysis in agricultural applications. Sharma [7] contributed valuable insights into random forest applications in agricultural settings, while Cortes and Vapnik [14] established the fundamental principles of support vector machines that are now widely used in FCR prediction.

Fu et al. [15] developed a comprehensive data collection and modeling framework specifically for feed efficiency prediction. LeCun [16] and Piles et al. [17] established the theoretical foundations for deep learning applications in agricultural settings. Shirzadifar [18] conducted a systematic review of machine learning applications in livestock farming, identifying key challenges and opportunities.

The integration of advanced technologies has further enhanced our capabilities. Johansen et al. [19] demonstrated the successful application of neural networks in chicken FCR prediction, Le Boucher et al. [20] were among the first to apply Bayesian methods for predicting the feed intake of seabass. Ellis [21] developed specialized deep learning architectures for precision farming applications.

Recent methodological advances include Monizet al.’s [22] work on Gradient Boosting for mixed models and Fahrurrozi’s [23] development of interpretable machine learning frameworks. Mishra et al. [24] made significant contributions by integrating IoT data with machine learning models, while Cravero et al. [25] combined genetic algorithms with machine learning for improved FCR prediction.

The field has seen rapid advancement in analytical capabilities. Davison et al. [4] optimized feed management through advanced predictive analytics, while Koppe et al. [26] expanded the applications of data mining in agricultural modeling.

Comprehensive reviews and methodological advances have shaped current understanding. Technical innovations continue to emerge, with Griesbach et al. [27] optimizing Gradient Boosting parameters for time series forecasting, and Konstantinov et al. [28] proposed a local and global black box model interpretation method based on a generalized additive model. It can be seen as an extension or modification of the algorithm using neural addition models. Cheng et al. [29] contributed to the methodological framework with their work on supervised learning and random forest applications.

Recent advances in modeling complex systems, demonstrated by Koppe et al. [26] and Kriegeskorte [30], have provided new tools for understanding FCR dynamics. Goodfellow et al. [31], Sutton and Barto [32], and Gu et al. [33] established fundamental principles for advanced machine learning applications. Benos [34] provided a comprehensive review of current applications, while Welper [35] contributed important insights into neural network training optimization. Certainly, the transformation of wearable devices also provides more comprehensive data collection methods for animal breeding and animal management [36,37].

In animal breeding programs, determining shorter feed intake measurement intervals and estimating their reliability is crucial for enhancing selection accuracy for target traits and reducing costs. This is particularly significant for finishing-stage pigs, as shorter feed intake measurement intervals require less single-space feeder time. With a fixed number of feeders, operations can test more batches of pigs. This study aims to evaluate the predictive reliability of individual feed conversion ratios (FCRs) using large-scale commercial feed intake records, specifically assessing the impact of different test intervals and starting points. Of particular interest are intervals where the starting body weight reaches at least 40 kg—a key transition phase from the early growing period to the rapid growth phase of finishing. Understanding pig growth phases is crucial for interpreting our findings. Pigs typically undergo distinct growth stages: early growth (weaning to ~40 kg), rapid growth phase (~40–90 kg), and finishing phase (~90 kg to market weight). The 40 kg threshold identified in our study corresponds to the transition from the early to the rapid growth phase, when feed conversion patterns become more stable and predictable.

This study aims to build upon these foundations by developing an integrated approach to FCR prediction. Using 438,552 feed samples from two core farms in Sichuan Province, we utilize nineteen machine learning algorithms to explore the feasibility of predicting long-term FCRs from short-term data. Our work synthesizes the methodological advances described above while addressing the specific challenges of FCR prediction in practical farming environments.

## 2. Materials and Methods

**Figure 1:**
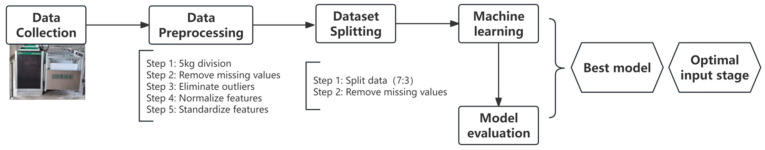
FCR experimental flowchart.

The basic experimental process is shown in Figure 1 below:

### 2.1. Animal and Data Collection

FCR data were collected from Osborne feed testing stations installed at two core breeding farms in Sichuan Province, China. Feeding data were collected using the same Osborne system model across both farms to ensure uniformity in methodology and contribute to consistent rearing conditions. Upon entry of each pig into the feed testing station, the system initiated the collection of data. The dataset comprises 438,552 records of individual feeding visits, encompassing data from 622 individual pigs. Each record includes animal ID, body weight at the time of visit, and feed intake during the visit. Pig body weights ranged from 20 to 180 kg.

### 2.2. Data Preprocessing

Prior to analysis, we performed a series of preprocessing steps to ensure data quality:

Step 1—Data cleaning and 5 kg division: The core steps of feed data aggregation were carried out using the daily time dimension while considering the continuity of individual pigs, which specifically included the following: (1) Data preprocessing and outlier removal: we took the feeding event of a single pig (each record of entering the feeding station) as the basic unit and removed unreasonable data through dynamic outlier detection, such as weight anomalies (marked as abnormal if the current weight deviated from the 3-day rolling median by more than 20% or 3 standard deviations) and feeding rate anomalies (defined as abnormal if the feeding rate exceeded 10 g/s), and the output was the cleaned single feeding event record. (2) Daily summary aggregation: grouping by animal_number and visit_date, calculating the daily average weight (daily_avg_weight) and the last value of the day (daily_last_weight) for weight, the sum of all feeding events on that day (daily_total_feed) for feed intake, the total duration of all feeding events on that day (daily_total_duration) for feeding time, the average value of all records on that day (daily_avg_feed_rate) for feeding rate, and the first recorded value (gender_first) for static attributes such as gender. (3) Trend verification (validate_animals function): further removing abnormal pigs by checking whether the weight dropped by more than 5% for two consecutive days and whether the feed conversion ratio (total cumulative feed intake/total weight gain) was lower than 2.0 (assuming FCR should not be lower than 2.0). For the distribution characteristics of feed samples, the original data contained 438,552 feeding event records (code input data), which were reduced to daily single-pig summary records after daily aggregation. Each summary record included 6 core indicators (average weight, last weight, total feed intake, total time, average rate, and gender). Finally, the data were aggregated according to the pig weight stage of 5 kg, and the large-interval FCR was calculated.

Step 2—Removing missing values: Rows with any missing values were removed from the dataset. This approach is effective when the proportion of missing data is small (5% in our dataset).

Step 3—Eliminating outliers beyond three standard deviations and normalization: The mean (*μ*) and standard deviation (*σ*) of the dataset were calculated. Features were normalized to place them on the same scale of measurement. The formula used was

(1) $$z=\frac{x-u}{s}$$

where *x* is the current sample value, and *μ* and *σ* are the mean and standard deviation of all samples, respectively.

Step 4—Feature standardization: Features were standardized by subtracting the mean and dividing by the standard deviation, resulting in a distribution with a mean of 0 and a standard deviation of 1. This enables optimization algorithms like gradient descent to converge faster [11]. Models such as SVM, Linear Regression, and logistic regression perform better on standardized data. Feature scaling can also reduce model training time by eliminating the influence of dimensionality. The formula used is

(2) $$y=\frac{x-x\;min}{(x\;max-x\;min)-1}$$

where *x* represents the sample value, *x min* and *x max* represent the minimum and maximum values of all samples, respectively, and *y* is the scaled value of the feature.

After quality control, the dataset showed a mean of 2.1090, a median of 2.1021, a maximum of 2.4114, a minimum of 1.7187, a standard deviation of 0.1330, and a coefficient of variation of 6.3049%.

These preprocessing steps are particularly important for distance-based algorithms (SVM, K-NN), gradient-based optimization (neural networks), and regularized linear models (Ridge, Lasso) which are sensitive to feature scaling.

### 2.3. Dataset Splitting

The data were divided into a training set and a validation set in a 7:3 ratio. To assess the impact of input FCR interval size on long-term FCR prediction, the validation set data were partitioned at intervals of 5 kg, 10 kg, 15 kg, 20 kg, 25 kg, 30 kg, 35 kg, and 40 kg.

### 2.4. Machine Learning Algorithms

In our study, we systematically compare a variety of machine learning models to assess their performance in handling both discrete and continuous data types. This comparative analysis involves nineteen distinct models, each chosen for its unique capabilities and theoretical underpinnings. These models are categorized based on their primary functions and methodologies.

We begin with fundamental linear models such as Linear Regression, Ridge Regression, Lasso, and ElasticNet. These models are chosen for their simplicity and effectiveness in linear approximation tasks [13]. Ridge and Lasso regression address issues of multicollinearity among independent variables, yielding more stable parameter estimates. Meanwhile, Lasso regression and ElasticNet achieve feature selection through penalty terms, which helps reduce model complexity and improve interpretability.

Additionally, we include Bayesian Ridge Regression, which incorporates Bayesian reasoning principles. This model provides a probabilistic approach to Linear Regression by offering uncertainty estimates for parameter estimates through posterior distributions, enabling the calculation of confidence intervals for predictions. By integrating prior distributions, Bayesian Ridge Regression effectively mitigates overfitting, particularly in scenarios with small datasets or high-dimensional feature spaces. Although Bayesian computations are generally more complex, the implementation of Bayesian Ridge Regression remains relatively straightforward and can be efficiently solved using existing computational tools.

In the realm of ensemble methods, our study incorporates random forest, Gradient Boosting, AdaBoost, Extra Trees, and specialized Gradient Boosting machines (LightGBM, XGBoost, and CatBoost). These models are known for their robustness and ability to improve predictive accuracy through techniques such as bagging and boosting.

We also examine traditional support vector machines, which are binary classification models that map input data to a high-dimensional space and find an optimal hyperplane for classification tasks. Furthermore, we explore kernel-based methods, including Support Vector Regression (SVR), NuSVR, and Linear SVR. These are crucial for their capacity to model non-linear relationships using different kernel functions.

For a non-parametric perspective, we utilize the decision tree model, valued for its interpretability and simplicity [35]. We also employ the K-Nearest Neighbors (K-NN) algorithm, which makes predictions based on the proximity to its neighbors.

Additionally, we investigate the Gaussian Process model, which offers insights into the uncertainty of predictions, and the Multi-Layer Perceptron (MLP), a basic form of neural networks. These models are evaluated for their effectiveness in more complex and high-dimensional spaces [16,17].

This comprehensive suite of models enables a thorough comparison and evaluation, allowing us to determine the most effective techniques for various types of data and modeling challenges.

The Gradient Boosting model, which exhibited the best performance in this study, is an ensemble model that combines multiple weak learners (usually decision trees) to create a strong learner. It builds the model in a stage-wise fashion, with each new tree attempting to correct the errors made by the previous trees. The model is trained using a differentiable loss function and gradient descent optimization [22]. These 19 algorithms were selected based on their diverse approaches to data modeling, ranging from simple linear models to complex ensemble methods, to comprehensively evaluate different machine learning techniques for FCR prediction.

Although the above algorithms are designed to directly handle continuous numerical inputs, in this specific study on feed conversion ratio (FCR) prediction, we preprocessed the continuous variable of body weight through binning (at 5 kg intervals), which made this feature appear as an ordered categorical variable (ordinal discrete data) when input into the model.

This binning preprocessing is primarily based on the following two considerations:

(1) Domain Practice and Interpretability: In livestock management and animal nutrition research, animal body weight stages (e.g., <5 kg, 5–10 kg, 10–15 kg, etc.) are typically closely associated with specific feeding strategies, nutritional requirements, and health management. Converting body weight into explicit stage categories facilitates the interpretability of model predictions (i.e., FCR values for specific weight stages) by domain experts and their application in practical production decisions.
(2) Feature Engineering and Mitigation of Potential Issues: Body weight data may exhibit skewed distributions or contain potential measurement outliers. Binning at 5 kg intervals can mitigate the impact of these issues and may help simplify the learning boundaries of certain complex models (such as tree-based ensemble methods). The superior performance of Gradient Boosting in this study partially validates the effectiveness of this feature representation in capturing the relationship between weight stages and FCR.

It should be noted that while the underlying algorithms can handle continuous data, our choice to bin weight data into 5 kg intervals was a deliberate preprocessing decision based on domain expertise and practical considerations in livestock management, where weight-based feeding stages are commonly used in commercial operations.

Hyperparameter optimization was performed using a grid search with cross-validation for all models to ensure fair comparison. The optimal hyperparameter configurations are detailed in Appendix A. For specific hyperparameter settings, please refer to Tables S1 and S2.

### 2.5. Model Evaluation

All analyses were performed using Python 3.8.18 with the scikit-learn 1.3.2 library. Four basic metrics were used to evaluate the models’ performance:

(3) $$\begin{array}{cc} R^{2}=1 & -\frac{\sum_{i=1}^{n}{\left(y_{i}-f_{i}\right)}^{2}}{\sum_{i=1}^{n}{\left(y_{i}-\overset{\_}{y}\right)}^{2}}\\ RMSE & =\sqrt{\frac{1}{n}\sum_{i=1}^{n}{\left(y_{i}-f_{i}\right)}^{2}},\\ MAE & =\frac{1}{n}\sum_{i=1}^{n}\left|y_{i}-f_{i}\right|,\\ MAPE & =\frac{1}{n}\sum_{i=1}^{n}|\frac{y_{i}-f_{i}}{y_{i}}|, \end{array}$$

where *n* is the number of samples in the data, and y_mean is the average of the samples.

Additionally, the Pearson correlation coefficient was used to evaluate the correlation between predicted and true values, indirectly reflecting the predictive performance of the model. It measures the linear relationship between two quantitative variables, requiring the data to be continuous and at least approximately normally distributed. The Pearson coefficient is sensitive to outliers. The formula is:

(4) $$r=\frac{\left(\sum (x_{i}-(\bar{x}))(y_{i}-(\bar{y}))\right)}{\surd (\sum (x_{i}-(\bar{x)})2*\sum (y_{i}-(\bar{y)})2}$$

## 3. Results

Having established our methodology, we now turn to the results of our analysis, which demonstrate the efficacy of our approach in predicting long-term FCR using short-term data.

### 3.1. Data Preprocessing Results

After handling missing values and performing data validation, individual data from 138 pigs with weights ranging between 30 and 105 kg were retained. The FCR was calculated for each 5 kg interval, as well as the overall FCR for the entire 30–105 kg range.

### 3.2. Model Training

**Figure A1:**
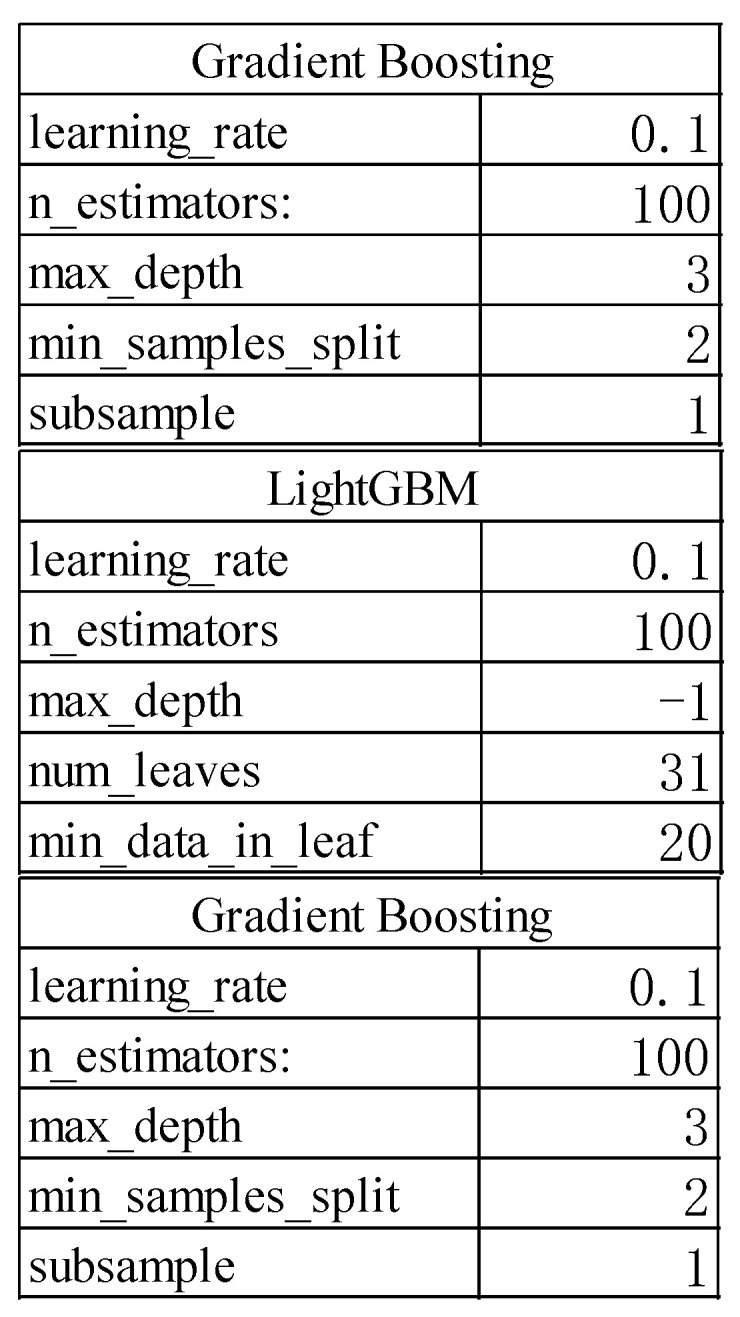
Model Training Parameter Settings.

Among the 19 models tested, Gradient Boosting, LightGBM, and CatBoost consistently outperformed others. Therefore, we focus on these three models for further analysis. The model training employed a grid search strategy for hyperparameter optimization to identify the best-performing configuration for each model. The optimal hyperparameters identified through this process are detailed in Appendix A Figure A1. For specific hyperparameter settings, please refer to Tables S1 and S2.

The model training results are presented in Table 1. Among the models tested, Gradient Boosting, LightGBM, and CatBoost achieved the highest performance, with R^2^ values around 0.5. The Gradient Boosting model demonstrated the best overall performance (R^2^ = 0.51, RMSE = 0.09, MAE = 0.07, MAPE = 0.03). The reason some other models performed poorly may be due to the relatively complex linear relationships in the data, which limited the effectiveness of models such as Linear Regression, Ridge Regression, and Lasso regression. Additionally, some models may suffer from overfitting, particularly in cases of high-dimensional data or insufficient feature selection, leading to their poor performance on the test set.

### 3.3. Model Prediction Performance Under Different Interval Sizes

The trained Gradient Boosting model was used to evaluate the impact of different FCR interval sizes on 30~105 kg FCR prediction, using the validation set (Table 2). When the interval reached 40 kg, the model achieved an R^2^ of 0.72, and the correlation between predicted and actual values reached 0.85.

The trained LightGBM model was also evaluated using the validation set (Table 3). When the interval reached 40 kg, the model achieved an R^2^ of 0.71 and a correlation of 0.84.

Similarly, the trained CatBoost model was evaluated (Table 4). When the interval reached 40 kg, the model achieved an R^2^ of 0.72 and a correlation of 0.85.

**Figure 2:**
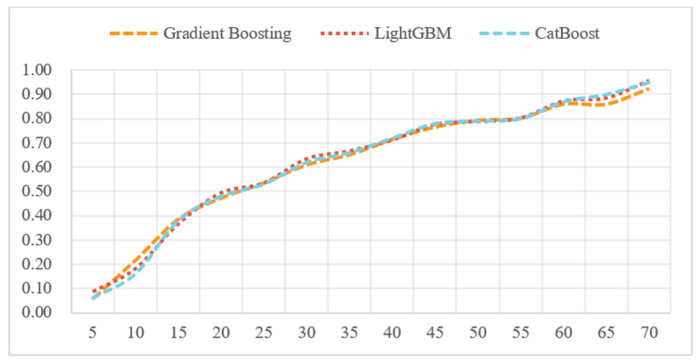
R^2^ curves for Gradient Boosting, LightGBM, and CatBoost models.

Figure 2 shows the R^2^ curves for the Gradient Boosting, LightGBM, and CatBoost models. As the prediction interval expands from smaller intervals (every 5 kg) to larger ones, the models’ R^2^ values improve significantly.

### 3.4. Optimal Prediction Interval with a Fixed Interval Size of 40 kg

Table 5, Table 6 and Table 7 present the prediction performance of the Gradient Boosting, LightGBM, and CatBoost models, respectively, for different starting weights while maintaining a consistent interval size of 40 kg. The analysis reveals that all three models demonstrate excellent predictive performance, particularly in the weight ranges of 50–90 kg and 65–105 kg.

Notably, the results indicate that measuring feed intake for pigs between 50 and 90 kg is most effective for predicting overall FCR in actual production scenarios. This finding has significant implications for optimizing data collection practices in livestock management.

## 4. Discussion

This study employs machine learning models to predict long-term (30–105 kg) FCR using short-term FCR data and provides a detailed evaluation of these models’ performance. The Gradient Boosting model outperforms all other models, achieving an R^2^ value of 0.72 when the prediction interval reaches 40 kg. Using data from the 50–90 kg and 65–105 kg weight ranges with a 40 kg prediction interval ensures the model’s accuracy and reliability.

### 4.1. Impact of Prediction Interval Size on FCR

The results demonstrate that as the prediction interval expands from smaller intervals (every 5 kg) to larger ones, the R^2^ values of the models improve significantly. For instance, from 5 kg to 40 kg, the R^2^ values of the Gradient Boosting model increase from 0.06 to 0.72. This indicates that larger prediction intervals provide more data aggregation, helping the model capture more stable and significant trends while reducing noise in the predictions.

Within smaller weight intervals, individual differences and random fluctuations may be more pronounced, potentially interfering with model predictions. In larger weight intervals, these short-term fluctuations are averaged out, allowing the model to better learn and predict long-term trends.

The study also observes that as the interval size increases, the correlation between predicted and actual values improves. This further confirms that larger intervals are more effective at simulating and reflecting actual growth and feeding responses during prediction.

**Figure 3:**
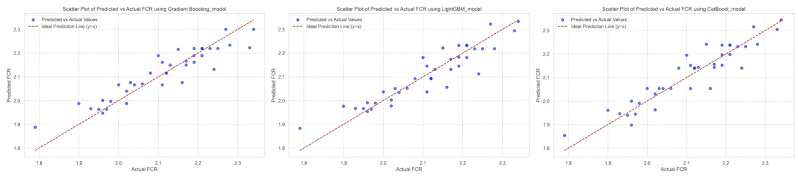
Predicted scatter plots of Gradient Boosting, LightGBM, and CatBoost models.

The horizontal clusters observed in Figure 3, particularly evident in the CatBoost model, are characteristic of tree-based ensemble methods. These horizontal clusterings result from the decision tree splitting logic and the discrete nature of our 5 kg weight interval binning, representing how tree-based algorithms partition the feature space rather than indicating model deficiencies.

### 4.2. Impact of Different Prediction Intervals with the Same Interval Size

The 50–90 kg and 65–105 kg weight ranges exhibit the best model prediction performance. Since the 65–105 kg range is already in the later stages of pig growth in this model, it has limited significance in actual production. In practice, measuring feed intake for pigs between 50 and 90 kg, which focuses on the mid-growth stage, is most important for animal feeding and pathological management. Accurate FCR prediction can significantly impact cost-effectiveness, particularly in pig farming. While our method demonstrates strong predictive power, it is important to note its limitations. The model’s performance may vary in different farming environments and with different pig breeds. Future studies should validate these findings across diverse settings.

### 4.3. Biological Factors Affecting FCR

The biological processes governing growth and feed efficiency can vary significantly across different weight ranges. In the 50–90 kg range, animals tend to be in a phase of rapid growth, where their metabolism is optimized for feed conversion. During this stage, the relationship between feed intake and weight gain is more linear and predictable, making it easier for machine learning models to learn from historical data. Conversely, outside this range, factors such as reduced growth rates, changes in dietary needs, or the onset of maturity may introduce more variability in FCR, making predictions less reliable.

### 4.4. Data Availability and Quality

The dataset utilized in this study likely reflects more consistent and comprehensive records for the 50–90 kg range. Data quality plays a crucial role in the performance of machine learning models. As animals grow, variations in feed composition, management practices, and environmental factors may lead to inconsistent data outside the 50–90 kg range. Therefore, models trained on data from this interval can achieve higher accuracy due to the inherent consistency in the growth dynamics and feeding practices observed within this weight category.

### 4.5. Practical Implications for Production

Understanding that FCR predictions are most reliable in the 50–90 kg range has significant implications for production practices:

Optimized Feeding Strategies: Producers can focus on implementing targeted feeding strategies during this critical growth phase to maximize feed efficiency. By closely monitoring FCR in this range, adjustments can be made to feeding regimens in real time, ensuring that animals receive the appropriate nutrients to maintain optimal growth rates.

Resource Allocation: With accurate FCR predictions, producers can allocate resources more effectively. This includes better planning of feed purchases and inventory management, ultimately leading to cost savings and reduced waste.

Predictive Management: By utilizing machine learning models for ongoing FCR monitoring, producers can establish predictive management practices that anticipate growth patterns. This proactive approach can mitigate issues related to underperformance or health problems that may arise outside the optimal weight range.

## 5. Conclusions and Future Directions

This study demonstrates the feasibility of using machine learning techniques, particularly the Gradient Boosting model, to predict long-term FCR based on short-term FCR data. The findings highlight the importance of appropriate data preprocessing, model selection, and interval size determination for optimal prediction performance. The insights gained from this research can guide precision livestock farming practices, leading to more efficient resource management and sustainable animal husbandry. Real-time analysis of short-term feed conversion ratio (FCR) data can be conducted through data recorded at feed testing stations. Livestock producers can immediately understand the current feed efficiency, and predict long-term FCR trends based on short-term changes. This allows for proactive management strategies, such as adjusting immunization programs or modifying feed management practices. To ensure the accuracy of FCR predictions, it is still necessary to regularly evaluate model performance and adjust model parameters based on feedback, as well as to remove any anomalous data. These findings have immediate practical applications for commercial pig production, enabling producers to optimize feed efficiency monitoring while reducing equipment occupancy time and costs.

However, this study has certain limitations: the dataset used is from a specific region, potentially lacking complete representation of the diversity within pig farming environments. Subsequent research could incorporate data from multiple sources to improve the generalizability of the models.

While the Gradient Boosting model performed best in this study, other advanced machine learning algorithms, such as deep learning models, could be explored to potentially further enhance prediction accuracy. Incorporating diverse farming environments and breeds into the model training will enhance its robustness when faced with new data. Additionally, algorithms such as Gradient Boosting, with their powerful feature learning capabilities, can identify key factors influencing FCR across different settings, thereby improving performance in varied scenarios. Furthermore, considering changes in farming conditions, such as feed types and husbandry methods, the application of transfer learning can effectively bolster the model’s generalization ability. Therefore, future research should further validate the model’s performance across different farming environments to ensure its broad applicability in real-world production settings. Model Optimization: Investigate hyperparameter tuning and ensemble methods to refine the existing models for better performance. To further advance the research in this domain and contribute to the enhancement of global animal husbandry practices, several promising avenues for future work can be pursued. One valuable direction is to examine the impact of various environmental and management factors on pig farming outcomes more comprehensively. This could involve potentially integrating IoT data for real-time analysis, which would enable a more dynamic understanding of how different elements in the farming environment interact and influence results. Another important aspect is the exploration of different algorithms. Future research can evaluate the effectiveness of a wider range of machine learning techniques, such as neural networks and support vector machines. By doing so, it becomes possible to identify which approaches yield the best results in diverse contexts, considering the unique characteristics and requirements of pig farming scenarios. By building upon the foundation established in this study through these kinds of explorations, future research can make significant contributions to enhancing the efficiency and sustainability of global animal husbandry practices. Ultimately, this would lead to better outcomes for both farmers and livestock, fostering a more productive and environmentally friendly agricultural sector.

## Figures and Tables

**Table 1:** Prediction performance of different models for 30–105 kg FCR.

Model	R^2^	RMSE	MAE	MAPE
Linear Regression	0.36	0.11	0.08	0.04
Random Forest	0.41	0.10	0.08	0.04
Gradient Boosting	0.51	0.09	0.07	0.03
SVR	0.22	0.12	0.10	0.05
Ridge	0.36	0.11	0.08	0.04
Lasso	0.00	0.13	0.11	0.05
ElasticNet	0.00	0.13	0.11	0.05
Bayesian Ridge	0.36	0.11	0.08	0.04
Decision Tree	0.07	0.13	0.10	0.05
AdaBoost	0.37	0.11	0.08	0.04
Extra Trees	0.27	0.11	0.09	0.04
K-Nearest Neighbors	0.42	0.10	0.08	0.04
MLP	0.42	0.10	0.08	0.04
Linear SVR	0.25	0.12	0.09	0.04
NuSVR	0.24	0.12	0.09	0.04
Gaussian Process	−3.81	0.29	0.09	0.04
LightGBM	0.51	0.09	0.07	0.03
XGBoost	0.46	0.10	0.07	0.04
CatBoost	0.50	0.09	0.07	0.03

**Table 2:** Prediction performance of the Gradient Boosting model for 30–105 kg FCR under different intervals.

	R^2^	Pearson_Corr.	RMSE	MAE	MAPE
5	0.06	0.29	0.13	0.11	0.05
10	0.22	0.47	0.11	0.09	0.04
15	0.39	0.63	0.09	0.08	0.04
20	0.47	0.70	0.10	0.08	0.04
25	0.54	0.74	0.09	0.07	0.03
30	0.61	0.78	0.08	0.07	0.03
35	0.65	0.81	0.08	0.06	0.03
40	0.72	0.85	0.07	0.05	0.02
45	0.77	0.88	0.06	0.05	0.02
50	0.79	0.89	0.06	0.05	0.02
55	0.80	0.90	0.06	0.04	0.02
60	0.86	0.93	0.05	0.04	0.02
65	0.86	0.94	0.05	0.04	0.02
70	0.93	0.98	0.04	0.03	0.01

**Table 3:** Prediction performance of the LightGBM model under different intervals.

	R^2^	Pearson_Corr.	RMSE	MAE	MAPE
5	0.09	0.31	0.13	0.10	0.05
10	0.18	0.44	0.11	0.09	0.04
15	0.37	0.61	0.10	0.08	0.04
20	0.49	0.71	0.10	0.07	0.04
25	0.53	0.74	0.09	0.07	0.03
30	0.63	0.80	0.08	0.06	0.03
35	0.67	0.82	0.08	0.06	0.03
40	0.71	0.84	0.07	0.05	0.02
45	0.78	0.88	0.06	0.05	0.02
50	0.79	0.89	0.06	0.05	0.02
55	0.80	0.90	0.06	0.04	0.02
60	0.87	0.93	0.05	0.04	0.02
65	0.88	0.94	0.05	0.03	0.02
70	0.96	0.98	0.03	0.02	0.01

**Table 4:** Prediction performance of the CatBoost model under different intervals.

	R^2^	Pearson_Corr.	RMSE	MAE	MAPE
5	0.06	0.28	0.13	0.11	0.05
10	0.16	0.42	0.12	0.09	0.04
15	0.38	0.62	0.10	0.08	0.04
20	0.48	0.70	0.10	0.08	0.04
25	0.53	0.73	0.09	0.07	0.03
30	0.62	0.79	0.08	0.07	0.03
35	0.66	0.81	0.08	0.06	0.03
40	0.72	0.85	0.07	0.05	0.02
45	0.78	0.88	0.06	0.05	0.02
50	0.79	0.89	0.06	0.05	0.02
55	0.80	0.90	0.06	0.04	0.02
60	0.8	0.93	0.05	0.04	0.02
65	0.90	0.95	0.04	0.03	0.02
70	0.95	0.97	0.03	0.02	0.01

**Table 5:** Prediction performance of the Gradient Boosting model for different starting weights with a 40 kg interval.

	R^2^	Pearson_Corr.	RMSE	MAE	MAPE
30–70	0.53	0.73	0.08	0.06	0.03
35–75	0.66	0.82	0.09	0.07	0.03
40–80	0.60	0.78	0.08	0.06	0.03
45–85	0.71	0.84	0.07	0.05	0.02
50–90	0.81	0.90	0.06	0.05	0.02
55–95	0.72	0.87	0.06	0.05	0.02
60–100	0.74	0.87	0.06	0.05	0.02
65–105	0.85	0.93	0.05	0.04	0.02

**Table 6:** Prediction performance of the LightGBM model for different starting weights with a 40 kg interval.

	R^2^	Pearson_Corr.	RMSE	MAE	MAPE
30–70	0.49	0.72	0.08	0.06	0.03
35–75	0.69	0.84	0.08	0.07	0.03
40–80	0.57	0.76	0.08	0.06	0.03
45–85	0.68	0.84	0.07	0.05	0.02
50–90	0.80	0.90	0.06	0.05	0.02
55–95	0.71	0.87	0.06	0.05	0.02
60–100	0.76	0.88	0.06	0.05	0.02
65–105	0.86	0.93	0.05	0.04	0.02

**Table 7:** Prediction performance of the CatBoost model for different starting weights with a 40 kg interval.

	R^2^	Pearson_Corr	RMSE	MAE	MAPE
30–70	0.50	0.73	0.08	0.06	0.03
35–75	0.68	0.82	0.08	0.07	0.03
40–80	0.59	0.77	0.08	0.06	0.03
45–85	0.70	0.85	0.07	0.05	0.02
50–90	0.81	0.90	0.06	0.05	0.02
55–95	0.71	0.87	0.06	0.05	0.02
60–100	0.78	0.88	0.05	0.04	0.02
65–105	0.87	0.93	0.05	0.04	0.02

## Data Availability

The original contributions presented in this study are included in the article; further inquiries can be directed to the corresponding author. Code availability for access: https://github.com/DeepBLUP/FCR-Predication.git (accessed on 3 June 2025).

## Supplementary Materials

The following supporting information can be downloaded at: https://www.mdpi.com/article/10.3390/ani15121773/s1, Table S1: “best_hyperparameters” shows the model hyperparameter settings; Table S2: “optimized_model_results” presents the pre-training results of the model during grid search.

## Appendix A

Model Training Parameter Settings In this study, the main parameter settings are as follows:

The selection of these parameters is based on the following theoretical and empirical considerations:

Learning rate: This parameter controls the step size of each iteration, typically ranging between 0.01 and 0.1 [27]. A rate that is too small may cause slow convergence, while one that is too large may prevent convergence to an optimal solution.

Model complexity: The number of iterations (n_estimators) or maximum tree depth (max_depth) limits the model’s complexity to prevent overfitting. Generally, shallower trees and fewer iterations are sufficient [28].

Tree growth control: Parameters such as the minimum number of samples required for leaf nodes (min_samples_split, min_data_in_leaf) and subsample ratio help control the growth of each tree. Appropriate settings can improve generalization.

Regularization: Parameters such as l2_leaf_reg can limit the size of leaf node output values and reduce the risk of overfitting.

To optimize these hyperparameters, we employed grid search and cross-validation techniques. This approach helps balance training errors and generalization errors [36]. The meticulous selection and optimization of these parameters allow the model to thoroughly learn the intrinsic patterns of the data while preventing the memorization of noise. The robust performance of the final model on unseen data indicates its excellent generalization capabilities.

In summary, this study introduces a novel method based on machine learning techniques to predict long-term feed conversion ratio (FCR) utilizing short-term FCR data. Empirical studies have demonstrated that this method can effectively improve the prediction accuracy of feed conversion rate, serving as a robust tool for precision livestock farming. We anticipate that this study will make a significant contribution to the sustainable development of animal husbandry practices.
